# Does the likelihood of malignancy in thyroid nodules with RAS mutations increase in direct proportion with the allele frequency percentage?

**DOI:** 10.1186/s40463-022-00611-8

**Published:** 2023-02-11

**Authors:** Thomas J. Hudson, Marc Philippe Pusztaszeri, Michael P. Hier, Veronique-Isabelle Forest, Ji-Wei Yang, Richard J. Payne

**Affiliations:** 1 Department of Otolaryngology – Head and Neck Surgery, Jewish General Hospital, McGill University, 3755 Ch. de la Côte-Sainte-Catherine Rd., Montreal, QC H3T 1E2 Canada; 2 nt of Otolaryngology – Head and Neck Surgery, McGill University Health Centre, Montreal, QC Canada; 3 Department of Pathology, Jewish General Hospital, McGill University, Montreal, QC Canada; 4 Division of Endocrinology, McGill University Health Centre, Montreal, QC Canada

**Keywords:** Thyroid cancer, Genetics, Cytology, Allele frequency, Thyroid nodules

## Abstract

**Background:**

Genomic testing has enhanced pre-surgical decision making for cytologically indeterminate thyroid nodules, but there remains uncertainty regarding *RAS* mutations. The addition of extra genetic alterations to previous driver mutation panels has been shown to improve predictive value. This study aims to evaluate the relationship between the mutant allele frequency (AF) and likelihood of malignancy in thyroid nodules with *RAS* mutations.

**Methods:**

A retrospective cohort review was performed evaluating patients with indeterminate cytology (Bethesda categories III, IV and V) and ThyroSeq® v3 testing demonstrating a *RAS* mutation, who underwent surgery. Univariate and multivariate regression analyses were used to evaluate relationships between AF, other genetic alterations, and malignancy.

**Results:**

Thirty-nine patients met criteria, 77% of the thyroid nodules (30/39) were found to be malignant. None demonstrated aggressive pathology. On univariate regression, there was no relationship between AF and likelihood of malignancy. There was, however, a significant correlation between AF and the rate of an additional genetic alteration. Multivariate analysis found a trend between *RAS*, a second genetic alteration and malignancy, but it did not reach statistical significance.

**Conclusions:**

There was no direct relationship between the level of allelic frequency in thyroid nodules expressing *RAS* mutations and the likelihood of malignancy. There was a statistically significant relationship between increasing AF and the presence of a second genetic abnormality, suggesting a possible progression from initial driver mutation and then a second genetic alteration prior to malignant transformation.

**Graphical abstract:**

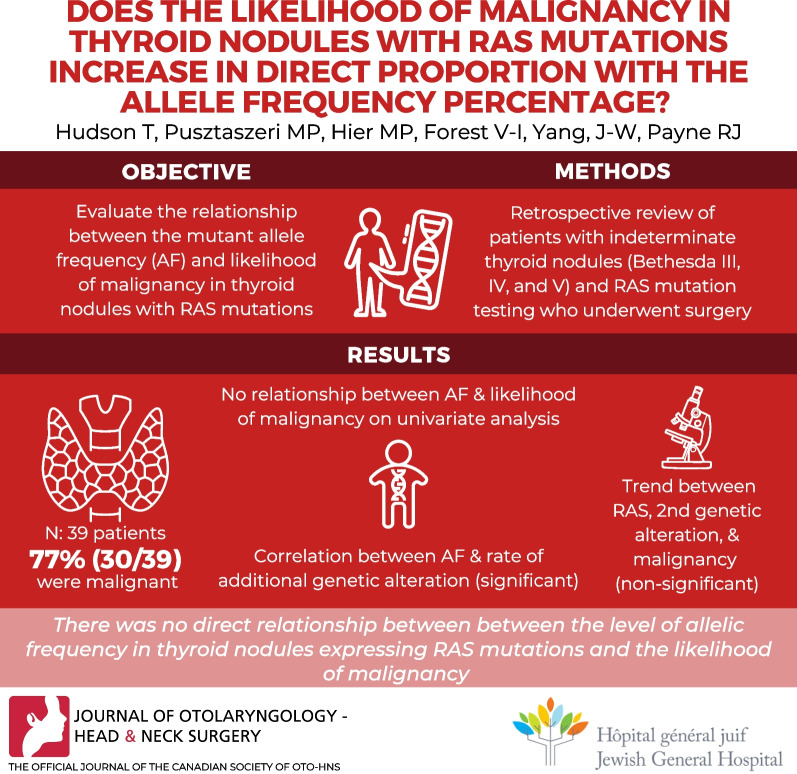

## Background

Thyroid cancer is currently estimated to be the 9th most common cancer diagnosis worldwide [1], with females being more frequently affected than males (lifetime risk of 1.7%) [2]. Thyroid malignancies are most often well differentiated and prognosis is overall excellent, with 5 year survival approaching 98% [2]. One of the current leading challenges in thyroid cancer care is accurate preoperative diagnosis.

Thyroid fine needle aspiration cytology (FNAC) by its nature is limited by a lack of tissue architecture and small volume sample size, and therefore often cannot definitively distinguish between benign and malignant thyroid pathologies. For this reason, the widely accepted Bethesda reporting system for thyroid FNAC [3] has been designed with six possible diagnostic categories to define the implied risk of malignancy: unsatisfactory (5–10%), benign (0–3%), Atypia of undetermined significance (AUS, 10–30%), follicular neoplasm (FN, 25–40%), suspicious for malignancy (SUS, 50–75%), and malignant (97–99%). Previous meta-analysis has shown that the three indeterminate categories (AUS, FN, SUS) represent between 20 and 25% of all thyroid FNAC results [4], leaving a challenging decision for many patients and thyroid specialists who must balance treating malignancy while avoiding unnecessary surgery in a nodule of uncertain nature.

To help improve pre-surgical diagnosis, several thyroid molecular tests are currently available. These include (with their estimated sensitivity and specificity (Sn, Sp)) the Afirma® gene sequencing classifier [5] (Sn 91%, Sp 68%), combination ThyGenNEXT® and ThyraMIR® [6] (Sn 95%, Sp 90%), and ThyroSeq® v3 [7, 8] (Sn 94%, Sp 82%). Most evaluate cancer risk by testing for known driver mutations—including *BRAF*, *NRAS*, *HRAS*, *EIF1AX* and *KRAS*. Additionally, some complement with their own genomic panel, evaluating other features such as gene fusions, copy-number alterations, and/or gene expression alterations. Although presence of the more common *BRAF* V600E driver mutations have been shown to be essentially diagnostic for thyroid cancer [9], *RAS* mutations are not quite as definitive as they can be found in the whole spectrum of follicular-pattern thyroid neoplasms including follicular adenoma, non-invasive follicular neoplasm with papillary-like nuclear features (NIFTP), follicular carcinoma, follicular variant of papillary thyroid carcinoma and poorly differentiated thyroid carcinoma. Therefore, *RAS* mutations are associated with a likelihood of malignancy ranging from 37 to 85% [10–12], resulting in a significant proportion of ultimately benign nodules subject to surgical resection (diagnostic lobectomy).

One of the molecular tests that is currently available, ThyroSeq® v3, reports a genetic quantity known as the mutant allelic frequency (AF), which is the proportion of mutant to normal DNA in a cytology sample [13]. It is noted to be a significant component in its cancer prediction algorithm, yet there is a lack of available data in the literature on the relationship between AF and the diagnosis of thyroid cancer. This data may be useful in better diagnosing and managing cytologically indeterminate thyroid nodules with *RAS* mutations. This study aims to evaluate specifically the relationship between the mutant allele frequency (AF) and likelihood of malignancy in thyroid nodules with *RAS* mutations.

## Methods

### Study design

A retrospective cohort study was performed evaluating patients with cytologically indeterminate thyroid nodules who underwent ThyroSeq® v3 testing from January 2017 to March 2020. The study was approved by the research ethics committees at the McGill University Health Centre and the Jewish General Hospital in Montreal, Quebec.

### Patient selection

Included patients were ≥ 18 years with at least one indeterminate thyroid nodule observed on cytology (AUS, FN, SUS), ThyroSeq® v3 testing demonstrating a *RAS* mutation (*NRAS*, *HRAS*, *KRAS*), and subsequently underwent thyroid surgery. Excluded patients were those awaiting thyroid surgery or with incomplete data at the time of collection.

The lead surgeon followed American Thyroid Association (ATA) guidelines [14] in the workup of said nodules. Patients with indeterminate nodules were presented the options of diagnostic lobectomy, thyroid molecular testing, or observation. They were encouraged to make the final decision based on their own preferences. In the event of indeterminate molecular results, the patient similarly was presented with diagnostic surgery or observation and encouraged to decide.

### Data collection

For each patient, the following data was reviewed: patient demographics, cytopathologic results, molecular test results, extent of surgery performed and results from final pathology. Genomic test results included the driver mutation, allele frequency and other genetic features when available. Note that ThyroSeq® divides other genetic features into either gene fusions (GF), copy-number alterations (CNA) or gene expression alterations (GEA).

Ultrasound guided FNA was performed by trained physicians with appropriate patient consent. Samples sent for genomic testing were prepared and shipped according to the provided ThyroSeq® protocol. All FNAC and surgical pathologic samples were analyzed internally by experienced thyroid pathologists, and were reported using the second edition of the Bethesda System for Reporting Thyroid Cytopathology [3] and the World Health Organization (WHO) classification of thyroid tumors [15], respectively. Pathologists were not blinded to molecular genomic test results.

### Statistical analysis

All statistical analysis was performed in R v4.0.2 (R Foundation for Statistical Computing, Vienna, Austria). The primary analysis aimed to evaluate relationship between allele frequency and malignancy in all *RAS* tumors, which was done using logistic regression. Subgroups based on *RAS* subtype *(HRAS, KRAS, NRAS)* were analyzed using logistic regression, and subgroups based on AF ranges (e.g. < 10%, 10–20%, etc.) were analyzed using a Chi-squared test. In a secondary analysis, both univariate and multivariate logistic regressions were used to evaluate relationship between AF and other genetic features (CNA, GEA), along with their combined risk of malignancy. These were the only pre-planned analyses; no others were attempted and omitted.

## Results

A total of 1066 surgical cases were screened between January 2017 and March 2020 inclusive, 39 of which met all criteria. Baseline characteristics, including Bethesda classification and *RAS* subtype, are shown in Table 1.

**Table 1:** Baseline Characteristic (*n* = 39)

Age (mean)	50.8
Male	7
Female	32
Bethesda category	
3 (AUS)	14
4 (FN)	21
5 (SUS)	4
*RAS* mutation	
*NRAS*	20
*HRAS*	18
*KRAS*	1
Surgical management	
Total thyroidectomy	5
Hemithyroidectomy	34

Final surgical pathology, shown in Table 2, demonstrated that 77% (30/39) of the *RAS*-positive nodules were malignant. There were three microcarcinomas that were found outside the sampled nodule (i.e. incidental), accordingly these nodules were counted as negative for malignancy. Of the remaining malignant cases, the majority were the follicular variant of papillary thyroid cancer (FV-PTC, 25/39), either fully encapsulated (4/39), with focal capsular invasion or partially encapsulated (13/39), or unencapsulated/invasive (8/39). The remainder were either the encapsulated solid variant of PTC (1/39) or encapsulated oncocytic (Hürthle) cell carcinoma with no vascular invasion (2/29). Note that there were none of the well recognized aggressive subtypes of PTC (e.g. Tall cell, hobnail/micropapillary, columnar cell, diffuse sclerosing) and none of the tumors had extra-thyroidal extension or other American Thyroid Association high risk features [14]. There were 10% (4/39) benign cases and 5% (2/39) NIFTP.

**Table 2:** Pathologic results

Diagnosis	*n*
Benign	7*
NIFTP	2
PTC – Follicular Variant	25
*Fully encapsulated*	*4*
*Partially encapsulated*	*13*
*Unencapsulated/invasive*	*8*
PTC – Oncocytic Variant	2
PTC – Solid Variant (encapsulated)	1
Hurthle Cell Carcinoma	2

*PTC* Papillary thyroid cancer, *NIFTP* Non-invasive thyroid neoplasm with papillary-like nuclear features. *Note that in three cases there was an incidental finding of microcarcinoma separate from the biopsied nodule, and therefore were considered benign

**Fig. 1:**
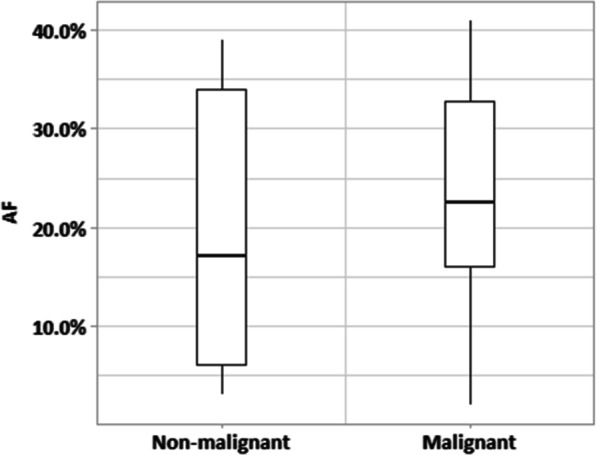
Distribution of allele frequency (AF) among studied tumors

The distribution of allele frequencies is shown in Fig. 1. The mean, median, and standard deviation of allele frequency in malignant cases were 23.2%, 23%, and 11%, respectively, and of non-malignant cases 20.1%, 17%, and 13%. In regression analysis, there was no significant relationship between allele frequency and malignancy in the full data set (*p* > 0.05). Dividing based on subtype of RAS (NRAS, HRAS) additionally did not show statistical significance between AF and likelihood of cancer. Subgrouping AF over the entire sample by value of < 10%, 10–19%, 20–29%, and 30 + % demonstrated a rate of malignancy of 57% (4/7), 80% (8/10), 86% (1/7), and 80% (12/15), respectively. Although the < 10% subgroup showed a lower rate of malignancy compared to the combined other cases (57% vs. 81%), this did not achieve significance in Chi-squared analysis (*p* > 0.05). The lowest AF in a malignant nodule was 2% and the highest AF in a benign nodule was 39%.

**Fig. 2:**
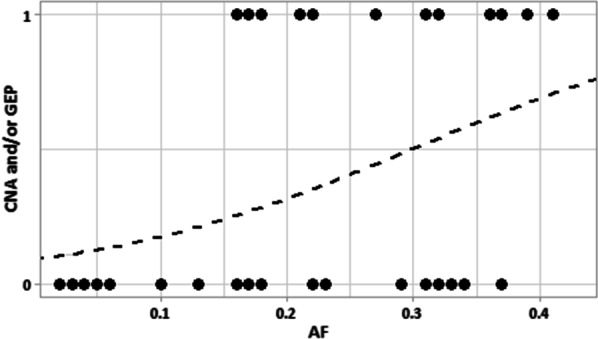
Presence of second genetic alteration in relation to allele frequency (*CNA* Copy-number alterations, *GEA* Gene expression profile alterations, *AF* Allele frequency). Dotted line represents probability fit by linear regression

On secondary analysis, evaluation of the relationship between driver mutation allele frequency and second genetic alteration (CNA and/or GEA) revealed a significant correlation (Odds ratio [OR] = 1.082, [Confidence Interval (CI) = 1.018–1.164], *p* = 0.0199). This is illustrated in Fig. 2, where there is a trend of increasing frequency of additional genetic alterations with increasing allele frequency. On multivariate analysis, there was a positive trend between second genetic alteration and malignancy (*r* = 1.97), but no statistically significant relationship was found between allele frequency, other genetic alterations, and cancer risk (all *p* > 0.05). Two of the nodules had a concurrent EIF1AX mutation, both of which were malignant. No other mutations were seen in this sample.

## Discussion

The *RAS* proto-oncogene encodes three highly homologous membrane-associated proteins: *NRAS*, *HRAS*, and *KRAS*. Under normal circumstances, these proteins are activated by the exchange of GDP for GTP by Grb2/SOS proteins, and then stimulate cellular pathways such as *Raf*-MAPK and PI3K-AKT. These pathways are known to be regulators of many fundamental cellular functions including growth and survival [16–19]. Certain point mutations on *RAS* genes, for example at the *NRAS* and *HRAS* codon 61, commonly implicated in *RAS*-associated PTC [16], result in inhibition of the GTP hydrolysis step required for *RAS* self-deactivation, and therefore leads to constitutional activation of the pathway. This mechanism has previously been shown to cause many forms of human cancer including pancreas, colon, lung, and differentiated thyroid cancer (DTC) [20, 21]. Although the *BRAF* V600E mutation is more common to DTC and essentially diagnostic for malignancy, *RAS* mutations remain less definitive, with a significant proportion of resected tumors yielding benign pathology or NIFTP [10, 16, 22].

Recent advances in thyroid genomic testing have gone beyond checking for presence of driver mutations alone, and now evaluate other genetic features including copy number alterations (CNA), single-nucleotide variants and alterations to the gene expression profile (GEA) [7]. The present study examined the allele frequency (AF), or the proportion of mutant DNA affected by the driver mutation to normal DNA, and evaluated its relationship to rate of malignancy among *RAS* tumors. In this sample, there was no significant relationship found between AF and thyroid cancer. This is in keeping with one previous analysis [23] where they categorically compared tumors with AF < 10% or ≥ 10% against presence of malignancy and did not find a relationship.

In the secondary analysis above, there was a significant relationship found when comparing *RAS* AF and presence of additional genetic alterations (CNA and/or GEA). Previous literature has suggested a stepwise *RAS*-mediated oncogenesis in which an initial driver mutation leads to further molecular alterations that promote development of initial cancer [16], and later de-differentiation into poorly differentiated [24] and anaplastic [25, 26] cancers. Indeed, the result above supports this notion, suggesting a possible progression starting with a first mutation (*RAS* driver), developing increased AF over time, and then acquiring a second alteration on a path to malignancy. Although the multivariate analysis could not confirm a relationship between additional genetic abnormalities and cancer, there was a positive trend in this relatively small sample, and a larger data set could evaluate this further.

Surgical pathology in this sample was largely in keeping with reports from the literature, where *RAS* mutations have been shown to be associated with thyroid neoplasms that are characterized by a follicular growth pattern, including follicular variant PTC. They additionally tend to be encapsulated and have a low probability of concerning histologic characteristics such as extrathyroidal extension, lymph node metastasis and vascular invasion [27–29], which again was seen in the present study. A few cases in our study were oncocytic or solid variant of PTC, but these cases were also mainly encapsulated and there is currently no evidence that these uncommon variants represent more aggressive tumors in the absence of other adverse features. This reinforces the idea that lobectomy or hemithyroidectomy would be an appropriate surgical choice for *RAS* positive tumors given proper pre-operative selection criteria (e.g. nodule < 4 cm, no radiotherapy history, clinically lymph node negative, etc.) [23].

Limitations of this study include the retrospective review of electronic medical records. It evaluated data from only two tertiary care centres in one city and would not account for geographic variations in thyroid cancer. Neither the surgeons nor the pathologists were blinded to results from cytology or genomic testing, which could introduce bias in decision making and analysis. The genomic test was mostly not covered by the government public health insurance plan, which likely added bias in patient demographics and selection. Lastly, the study was limited to surgical cases only, and therefore did not account for *RAS* tumors that were not resected.

## Conclusions

In this study, there was no direct relationship between the level of allelic frequency and the likelihood of malignancy in a sample of cytologically indeterminate thyroid nodules with a *RAS* driver mutation. There was a statistically significant relationship between AF and the presence of a second genetic abnormality (CNA and/or GEA), suggesting a possible progression from initial driver mutation, increasing AF, and then a second genetic alteration prior to malignant transformation.

## Data Availability

All data analyzed during the current study are available from the corresponding author on reasonable request.

## Abbreviations

AF: Allele frequency
FNAC: Fine needle aspirate cytology
AUS: Atypia of undetermined significance
FN: Follicular neoplasm
SUS: Suspicious for malignancy
Sn: Sensitivity
Sp: Specificity
NIFTP: Non-invasive follicular neoplasm with papillary-like nuclear features
CNA: Copy-number alterations
GEA: Gene expression alterations
WHO: World health organization
PTC: Papillary thyroid carcinoma
FV: Follicular variant
DTC: Differentiated thyroid cancer
